# Brenda Milner: Pioneer of the Study of the Human Frontal Lobes

**DOI:** 10.3389/fnhum.2021.786167

**Published:** 2022-01-03

**Authors:** Bryan Kolb

**Affiliations:** Department of Neuroscience, University of Lethbridge, Lethbridge, AB, Canada

**Keywords:** frontal lobe, prefrontal cortex, Milner, memory, temporal organization of behavior

## Abstract

Although the behavioral effects of damage to the frontal lobes date back to at least the late 19th century even midway through the 20th century very little was known about human frontal lobe function and there was a general consensus that the frontal lobe did not play a key role in cognition. This all changed when Brenda Milner published a chapter in a 1964 volume entitled: *The Frontal Granular Cortex and Behavior*. Milner’s chapter, “Some effects of frontal lobectomy in man,” was the first systematic study of the effect of frontal lobe excisions on cognition in human patients. Milner had access to a unique population of frontal excision patients at the Montreal Neurological Institute that were being treated by Wilder Penfield and his associates for a wide range of neurological disorders, including intractable epilepsy. Milner and her colleagues engaged in a more than 50-year study that has had a formidable impact on our understanding of frontal lobe function. Paralleling studies of frontal lobe function in non-humans they influence on understanding the evolution and function of the prefrontal cortex of mammals. Thus, although Brenda Milner is best known for her studies of human memory, she has had an equally important contribution to our understanding of the frontal lobes.

## Introduction

Brenda Milner is a pioneer in the development of the study of neuropsychology. She is best celebrated for her studies of memory changes in patients with cerebral injuries. She is also known for important discoveries on the nature of cerebral asymmetry in cognitive function. Nevertheless, her major contributions to our understanding of frontal lobe function are the foundation for how the frontal lobes are understood today. This paper briefly reviews her history, her work on memory and cerebral asymmetry, and her work on the human frontal lobe.

## Brenda Milner’s Research History

Raised in Manchester, England, Brenda entered Cambridge in 1936 to study mathematics but later switched to psychology. During World War II she worked in a radar establishment before moving to Montreal in 1944 with her husband, Peter Milner, who was unexpectedly suddenly sent there to help set up Canadian atomic-energy research. She got a job at the University of Montreal teaching psychology and animal behavior (in French). Following the war she began graduate work at McGill with Donald Hebb.

Brenda began working with Wilder Penfield’s neurosurgical patients in 1950 where she studied the role of the temporal lobes in visual perception and memory for her Ph.D. Penfield was a pioneer in the surgical treatment of intractable epilepsy and so Brenda was able to compare the effects of left and right temporal lobe resections. She and Penfield studied two patients who had unilateral temporal lobectomies, including the hippocampus, but unlike Penfield’s other patients these two patients had severe memory problems. She and Penfield suspected that these two patients might have had undetected damage in the unoperated lobe, leading to the severe memory loss (This was confirmed many years later from postmortem examination of one of the patients).

William Scoville, a neurosurgeon in Hartford Connecticut, learned about Penfield and Milner’s patients and suggested that they study one of his patients who had received a bilateral medial temporal resection and was severely amnesic. This opportunity led to Brenda’s now classic studies of patient H.M (Scoville and Milner, 1957). She was not only able to demonstrate the role of the hippocampus in memory but to show that there were at least two independent memory systems in the brain and, later in other patients, to show a complementary specialization of the left and right temporal lobes on processes such verbal vs non-verbal memory (e.g., Milner et al., 1968).

The asymmetry of memory functions was consistent with her earlier work on sensory perception and the temporal lobes and the idea of complementary specialization of the two hemispheres in the human brain remains a dominant theme throughout her research career. One important opportunity of studying Penfield’s patients was that there were also patients with frontal lobe resections, although there were far fewer frontal lobe patients than temporal lobe patients, so it took longer to develop a story regarding frontal lobe functions.

## Research on Frontal Lobe Functions Before Brenda Milner

There were many descriptions of the effects of frontal lobe injuries in patients dating back to the late 1800s, but most of these patients had large injuries or tumors and the symptoms observed were highly variable and not clearly related to the size, type, or location of the tumors (e.g., Hecean and Ajuriaguerra, 1956). This led to extreme views in the 1930s and 1940s of the importance of the frontal lobes in the “highest” cognitive functions such as intellectual synthesis, capacity for ethical behavior, control of affect, awareness of self, and recent memory (for a review, see Teuber, 1964). In contrast, later research began to deny any special importance of human prefrontal structures in cognitive functions (e.g., Hebb, 1945; Teuber et al., 1951). For example, Hebb proposed that the frontal lobes were necessary for the development of a cognitive framework underlying intelligence and aligned skills, but once a certain level was attained, the frontal lobes played a minor role. Thus, he noted that whereas large frontal lesions in children had marked effects on intelligence, similar injury in adulthood had little effect on IQ.

Teuber et al. (e.g., Semmes et al., 1955) did extensive studies of World War II and Korean war veterans with head injuries, but the focus was on sensory and perceptual functions rather than cognition, *per se*. An important aspect of Teuber’s studies was that it allowed him to propose a unitary function of the frontal lobe. Thus, he advanced a hypothesis that would tie together the diverse symptoms of frontal lobe injury. He proposed that it was related to “corollary discharge,” whereby the frontal lobe sends a signal to posterior cortex that presets the sensory regions to anticipate a motor act (Teuber, 1964). For Teuber, this mechanism was necessary for the brain to produce voluntary movements, as opposed to automatic reactions. He also suggested that corollary discharge could be necessary for more complex tasks, including cognitive tasks, although he was vague on how this might work. In short, both the absence of effects of frontal injuries on intelligence and the search for a unifying theory of frontal lobe function were historically important.

## Frontal Granular Cortex and Behavior, 1962

Brenda Milner’s studies in the 1960s that frontal lobe function was much more interesting than was appreciated and her discoveries redefined our understanding of the frontal lobes. Brenda Milner’s opportunity to introduce her work on frontal lobe came at a large symposium on the Frontal Granular Cortex and Behavior held at Pennsylvania State University in 1962, with the chapters being published somewhat later. Presentations at this symposium were from researchers from North America and Europe and included six on the human brain and 14 on rodent, carnivore, and primate brains.

Brenda Milner’s chapter differed from the other human-based chapters, which were mostly on patients with tumors, vascular accidents, or head injuries, and was similar to the non-human chapters in that her patients had circumscribed cortical excisions. When Brenda Milner began to study patients with surgical resections of the frontal lobe at the Montreal Neurological Institute (MNI), she was aware of ongoing studies on the effects of frontal lesions in non-human primates and carnivores and could see the importance of cross species comparisons. In particular, such studies consistently showed that monkeys, dogs, and cats with frontal lesions were impaired at reversal type tasks in which animals learned a rule that was suddenly changed and required a shift in response strategy (e.g., Warren, 1964). Brenda Milner was aware of a task that David Grant at the University of Wisconsin had invented as a human analog of reversal learning (the Wisconsin Card Sorting Task), which was inspired by work with monkeys by Harry Harlow who was also at Wisconsin. She obtained the task from David Grant and used it demonstrate a new role for the frontal cortex.

In the Wisconsin Card Sorting Task, the participants were presented with a deck of cards with multiple copies of cards that had one to four different designs (circles, triangles, stars, or crosses) in different colors (red, yellow, blue, and green). Four different cards are placed in front of a participant. They had different designs: a red triangle, two green stars, three yellow crosses, and four blue circles. A deck of 128 cards is presented and the task is to place each card from the deck in front of the appropriate card in the row, sorting by one of the three possible categories (color, number, and shape). The participants were not told how to sort and were only given feedback as “correct” or “incorrect.” Once a participant selected the correct category 10 consecutive times, the correct solution changed without warning. The task continued until the participant successfully completed six categories or used all 128 cards, whichever came first.

When Brenda gave the task to patients with dorsolateral frontal-lobe lesions the results were stunning (Milner, 1963, 1964). The task was presented prior to the surgical excision and then again postoperatively (see Table 1). The dorsolateral frontal lobe patients showed a small, non-significant, impairment prior to surgery but were severely impaired following surgery. In contrast, patients with orbitofrontal, temporal, or posterior cortical lesions performed as well as normal controls.

**TABLE 1:** Effects of frontal lobectomy on card sorting.

**Locus of excision**	**Number of categories**		**Total errors**	
Dorsolateral frontal	Preop: 3.3	Postop: 1.4*	Preop: 54.9*	Postop: 73.2*
Control:				
Orbitofrontal + temporal	Preop: 5.3	Postop: 4.9	Preop: 24.7	Postop: 27.6
Posterior cortex	Preop: 4.5	Postop: 4.6	Preop: 39.8	Postop: 31.0

*Data from Milner (1964). *p* < 0.05 or better.*

Two conclusions can be drawn from these results. First, the performance of the dorsolateral frontal group was in sharp contrast to the other groups in that the dorsolateral group demonstrated a clear deficit in cognitive function. Second, the contrast between patients with dorsolateral and orbitofrontal lesions suggested a differentiation of function within the frontal lobes. Such a differentiation had not been described before and it demonstrated that the frontal lobe did not have unitary function as had been proposed by Teuber. The Wisconsin Card Sorting Test remains a standard test of frontal lobe function today, although now it is often given on a computer screen. It is worth noting another feature this study, and all of Brenda’s studies, is that she has been able to provide diagrams of the excisions in each patient. There were no CT-scans or MRIs in the 1960s and early 1970s so previous studies by others could not easily speak to brain-behavior correlations. X-ray images do not identify details of brain injury so the surgeon-produced drawings of the excisions were crucial to her studies.

One of Brenda’s strong research qualities is her observational skills. She noted that although Penfield’s frontal lobe patients had normal intelligence and were not aphasic, they were lacking in spontaneous speech even though their word knowledge was normal. This suggested to Brenda that word knowledge and word use were dissociable abilities. When she gave the patients word fluency tests, such as the Chicago Word Fluency test, in which patients were asked to write down as many words starting with a particular letter, S for example, in 5 min, patients with left frontal removals produced only about 50% as many words as patients with right frontal or left temporal lesions (Table 2; Milner, 1964). This profound loss of verbal fluency was clearly evident in a left frontal patient that I studied at the MNI, who was a professor of botany with a verbal superior IQ. He produced very few words and after a few minutes of silence remarked “I can’t think of anymore.”

**TABLE 2:** Effects of frontal lobectomy on verbal fluency: Word fluency vs verbal recall.

**Locus of excision**	**Mean IQ**	**Word fluency**	**Verbal recall**
Left frontal	115.3	35.4*	18.9
Right frontal	108.0	56.8	17.1
Left temporal	115.3	57.6	12.4*

*Data from Milner (1964). *p* < 0.05 or better.*

As shown in Table 2, Brenda also gave the patients a verbal memory test and whereas the left frontal patients had low verbal fluency, their verbal recall was normal, which contrasted with left temporal patients who had normal fluency but impaired verbal recall. But the other important finding was that right frontal patients were normal at both tasks. The importance of the finding that left but not right frontal patients had low verbal fluency showed that there was an asymmetry in frontal lobe function.

**FIGURE 1:**
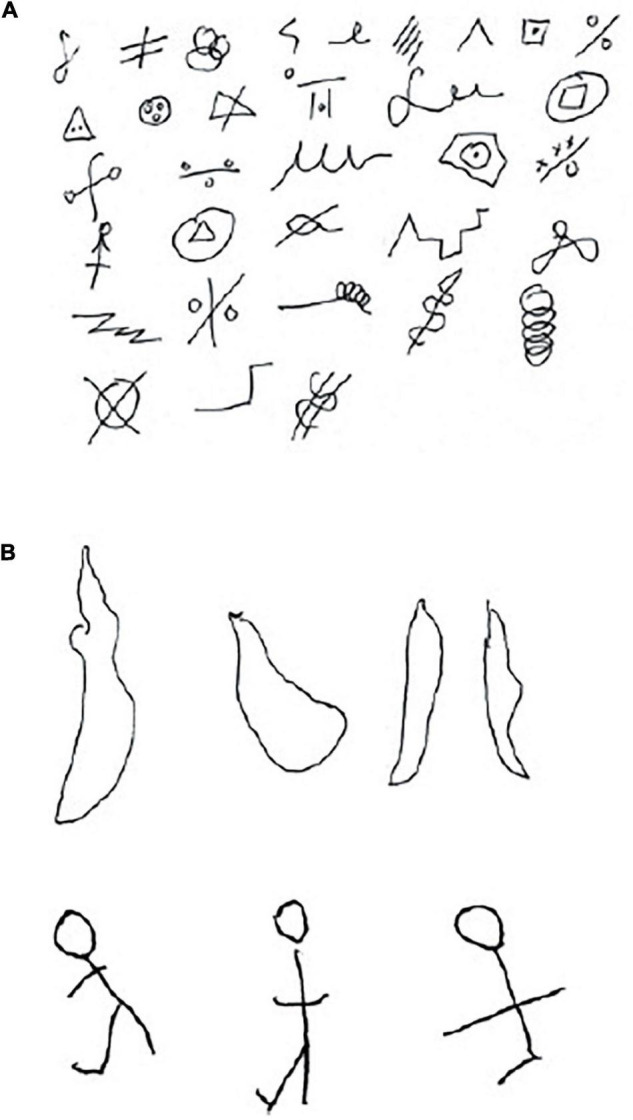
Design fluency. A control and frontal-lobe patient were asked to draw as many non-representational doodles as they could in 5 min. The control produced a diverse array of images. After several minutes the patient had produced only 4 drawings, all similar to each other, and so was encouraged to draw something else. She then drew three stick drawings (After Kolb and Whishaw, 2021). **(A)** Control. **(B)** Frontal lobe patient showing perseveration, lack of spontaneity, and rule breaking.

But if the right frontal lobe was not involved in verbal fluency, what might the complementary specialization be? A study devised by Jones-Gotman and Milner (1977) showed that right frontal lobe patients were impaired at a non-verbal analog of verbal fluency. Patients were asked to doodle by drawing as many designs as they could in 5 min. The drawings were supposed to be non-representational but spontaneous, much like students might draw in the margins of their notes during a lecture. The right frontal patients showed an impoverished output as illustrated in Figure 1. Not only did the patients have a paucity of designs but they often broke the rules and drew representational designs (such as the stick people in the figure) and also tended to perseverate with similar designs.

**FIGURE 2:**
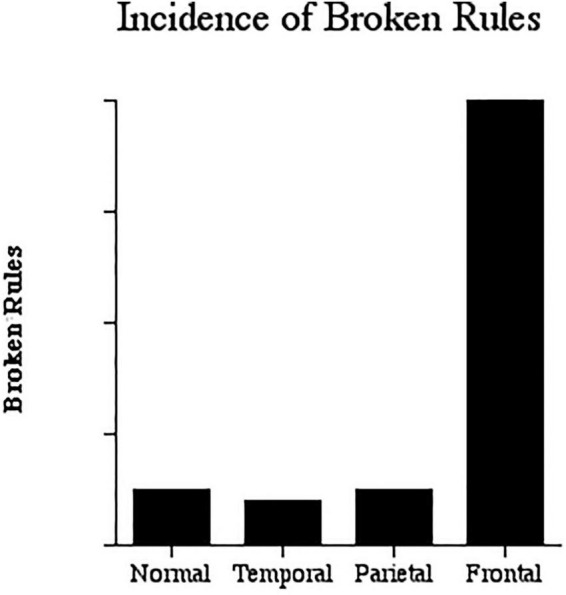
Broken Rules. The incidence of broken rules in stylus maze learning. The frontal lobe patients broke the rules far more frequently than the controls or other patients (Data from Milner, 1964).

Rule breaking by frontal lobe patients was also apparent in another study by Brenda Milner. She trained participants on a stylus maze task in which a board with 100 nail heads arranged in 10 rows of 10 was presented. The task was to learn the path from the bottom left corner to the top right corner by touching the stylus to the nail heads and making only vertical or horizontal moves. Every time the person deviated from the correct path, an error counter clicked noisily, thus informing the person of an error. One important rule for the test was that if a person made an error, they had to return to the preceding nail head and must not move diagonally but only upwards or laterally. As Figure 2 illustrates, frontal lobe patients consistently broke the rules (see also Milner, 1965).

In sum, the Milner (1964) chapter in the *Frontal Granular Cortex and Behavior* made these novel observations: (1) Although IQ was normal, frontal lobe patients had clear, and previously unknown, cognitive deficits; (2) The effects of dorsolateral prefrontal and orbital frontal lesions could be dissociated; (3) The effects of right and left frontal lesions could be dissociated – thus there was complementary specialization; (4) Patients with frontal lobe lesions did not follow rules (this turns out to be true not just in the performance of neuropsychological tests but in life in general); and (5) By studying the MNI population it was possible to conduct studies that could be directly compared to those on laboratory animals with focal lesions in the same regions.

## Effects of Frontal Lobe Lesions in Humans 1970–2004

Brenda Milner’s pioneering studies of the 1960s described above were merely a beginning and were followed by 4 decades of additional studies featured in 23 papers (see Table 4). These studies can be categorized into seven types of studies summarized in Table 3. A few examples will serve to illustrate.

**TABLE 3:** Effects of frontal lobectomy on maze learning.

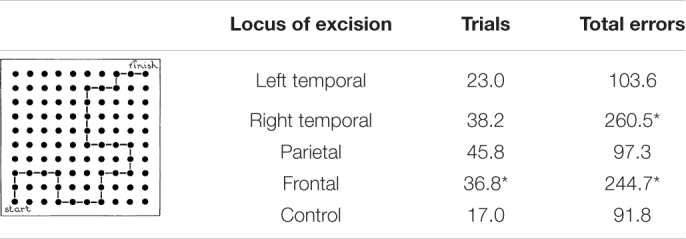

*Data from Milner (1964). *p* < 0.05 or better.*

**TABLE 4:** Studies of Human frontal lobes function by Milner and colleagues: 1970–2004.

**Category**	**Example publications**
Face and limb motor control	Kolb and Milner, 1981; Leonard et al., 1988a, b; Leonard and Milner, 1991a, b
Interference effects on memory	Della Rocchetta and Milner, 1993; Smith et al., 1995
Recency memory	McAndrews and Milner, 1991; Milner et al., 1991
Episodic memory	Kohler et al., 2004
Working memory	Frisk and Milner, 1990; Pigott and Milner, 1994; Leonard and Milner, 1995
Spontaneity of behavior	Jones-Gotman and Milner, 1977; Kolb and Milner, 1981
Cognitive Estimation	Smith and Milner, 1984, 1988
Cognitive risk taking	Miller, 1985*; Miller and Milner, 1985
Temporal organization of behavior	Petrides and Milner, 1982; Alivisatos and Milner, 1989
Language	Klein et al., 1995
Social cognition	Kolb and Taylor, 1981 *
Associative learning	Petrides, 1985 *

*Studies that were done with Brenda Milner but she is not an author.

In a classic paper Lashley (1960) asked how movements are put together in a particular order. How is it, for example, that a pianist can play a piece so quickly and accurately. Each note or movement could not be “thought of” separately because it would take the brain too long to confirm that each was completed before moving on to the next. Lashley suggested that the function of serially ordering complex chains of movement must reside in the neocortex – but where?

**FIGURE 3:**
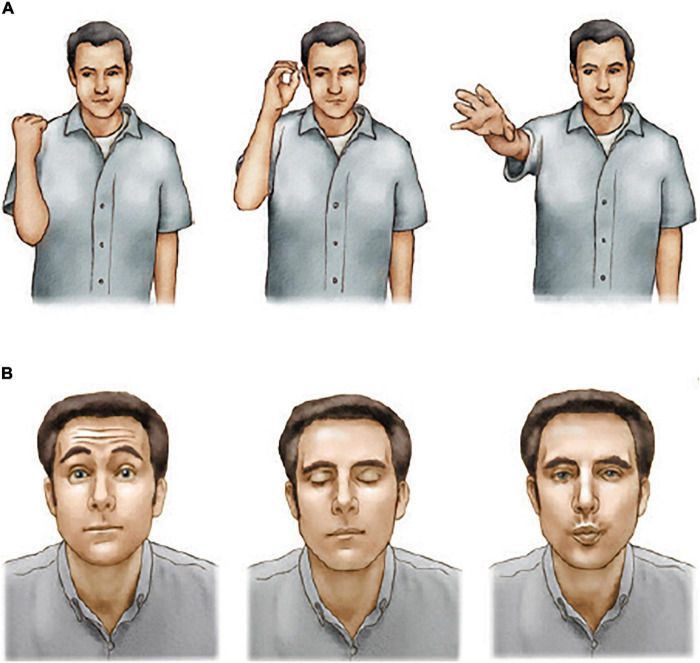
Arm and facial movement copying. Participants were asked to copy the arm or facial movements immediately after seeing them. Frontal lobe patients made errors on both tests but were especially impaired at the face copying test (After Kolb and Whishaw, 2021). **(A)** Serial arm-movement copying test. **(B)** Serial facial-movement copying test.

Kolb and Milner (1981) asked patients to copy a series of arm or facial movements (see Figure 3). Patients with frontal lobe lesions had an impairment although the impairment was much larger in left parietal lobe patients. In contrast, patients with left or right frontal lobe lesions were severely impaired at copying facial movements even though they able to copy the individual movements. Analysis of the errors made by the frontal lobe patients showed two main error types: (1) a failure to put the movements in the correct order; and (2) a failure to remember the items. These errors suggest that the deficit in serial ordering was in movement programming as well as working memory. A greater deficit copying face vs arm movements suggests that the frontal lobe may play a special role in controlling face movements, possibly because of the role of sequences of facial movements in talking, singing, and facial expressions.

The sequencing deficit could also be interpreted as a deficit in the temporal organization of behavior as later shown by Milner et al. (1991). Milner et al. (1991) presented participants with a deck of 184 cards on which there were two concrete words (e.g., greyhound, schoolboy) in three different tests. First, the participant read the words aloud and turned to the next card. Whenever a card appeared bearing two words separated by a question mark, the task was to identify which word was read most recently. In this test of recency, both cards had been seen before (e.g., 8 items vs 32 items ago). In the second condition, one of the words was novel. In this case the task became one of recognition memory, rather than recency memory. In the third condition, the test the words were replaced either by representational drawings (e.g., a bird, a lamp) or abstract-paintings providing non-verbal tests of recency and recognition that mirrored the verbal tests.

**FIGURE 4:**
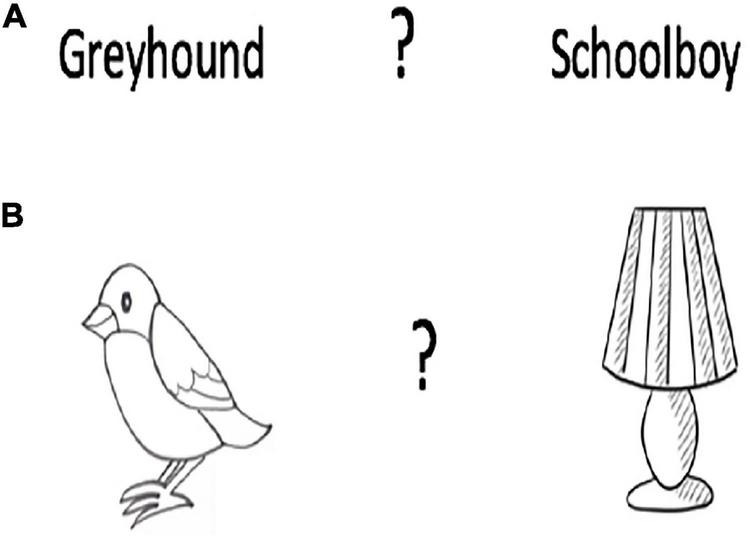
Recency memory. Participants were shown a stack of a deck of on which there were two concrete words or two representational drawings. The participant must point to the word or drawing seen most recently. Frontal lobe patients were impaired at identifying the most recently seen item. **(A)** Verbal. **(B)** Non-verbal.

Patients with left or right frontal lesions were normal at item recognition but impaired at the recency discrimination for the concrete words relative to controls or patients with temporal lesions. Patients with right but not left frontal lesions were impaired at recency discrimination, but not item recognition, on both of representational drawing and abstract drawing tasks (see Figure 4). Once again, this study confirmed Brenda Milner’s discovery of hemispheric asymmetry related to the nature of the stimulus material, as well as showing a role of the frontal lobe in recency memory judgments. In addition, analysis of performance deficits and lesion location revealed that damage to the left mid-lateral frontal region produced the largest deficits in verbal recency judgments, once again supporting Brenda’s earlier studies showing regional specialization within the human frontal lobes. The special role of the mid-dorsolateral frontal cortex in serial order memory was later confirmed in studies of monkeys with circumscribed mid-dorsolateral lesions vs more posterior frontal lesions (Petrides, 1991).

**FIGURE 5:**
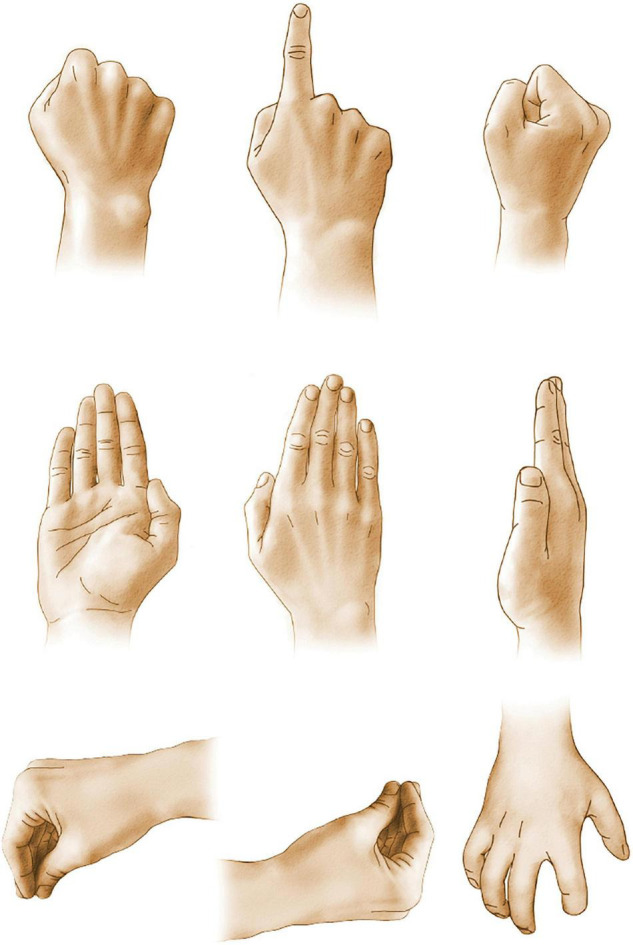
Associative learning. Participants had to learn to associate each hand posture with one of the nine colors and to perform the appropriate movement in response to the presentation of a color. Frontal lobe patients were impaired at associative learning.

Finally, a series of studies by Milner and Petrides (1984) and Petrides (1985, 1990, 1997) demonstrated a severe impairment on conditional associative tasks requiring the learning of arbitrary associations between a set of stimuli and a set of responses. For example, Milner and Petrides (1984) faced participants with six white cards and six blue lamps. When on lamp was turned on, the participant was to touch the cards individually until the correct one was found as the lamp was turned off. Over trials the task was to learn the association between each light and a card. Patients with either left or right frontal excisions showed marked impairments that contrasted with the normal performance by patients with left or right temporal lobe excisions. In an extensive series of studies Petrides (1997) asked patients to learn associations between nine colors and the nine hand postures as illustrated in Figure 5. Once again, patients with left or right frontal excisions were impaired at learning the conditional association.

## The Lasting Impact

As we reflect on the impact of Brenda Milner’s remarkable findings on frontal lobe function in humans we can point to several game-changing discoveries: (1) the frontal lobe plays a major role in cognitive processing; (2) the frontal lobe does not have a unitary function but has a wide range of cognitive and motor functions; (3) there is a differentiation in functions of different frontal lobe regions; (4) there is a complementary specialization of the left and right frontal lobes; (5) there are striking similarities in the effects of frontal lobe lesions in humans and laboratory animals, and monkeys in particular; and (6) although frontal lobe functions can be dissociated from posterior parietal and temporal lobe cortical functions, there is some overlap, which likely reflects the frontal lobe’s integration in extended cortical networks, and especially with the posterior parietal cortex.

In the past two decades there have been fewer studies on the effects of frontal lobe excisions on neuropsychological performance, as lesion studies are slowly being replaced with non-invasive imaging studies studying cerebral functions. But there is little doubt that the Brenda Milner studies of excision patients at the MNI has guided our thinking on what functions to further investigate with non-invasive imaging (e.g., Klein et al., 1995; Champod and Petrides, 2007; Kostopoulos et al., 2007). But imaging studies have their limitations, in part because of the methodological constraints incumbent with using techniques such as functional Magnetic Resonance Imaging, Positron Emission Tomography, Magnetic Resonance Spectroscopy and EEG. Furthermore, as noted by Vaidya et al. (2019) studies of humans with focal brain damage provide vital information about brain function that are distinct from imaging studies.

Over the coming decades it will take a combination of techniques, including lesion studies, imaging, and more molecular studies with laboratory animals to develop converging hypotheses on the nature of brain and behavioral functions. Brenda Milner’s pioneering studies provide us with novel insights into the frontal lobes and behavior and will continue to be invaluable to those that are built on hers.

## Author Contributions

The author confirms being the sole contributor of this work and has approved it for publication.

## Conflict of Interest

The author declares that the research was conducted in the absence of any commercial or financial relationships that could be construed as a potential conflict of interest.

## Publisher’s Note

All claims expressed in this article are solely those of the authors and do not necessarily represent those of their affiliated organizations, or those of the publisher, the editors and the reviewers. Any product that may be evaluated in this article, or claim that may be made by its manufacturer, is not guaranteed or endorsed by the publisher.
